# Effect of Grain Size and Porosity/Binder Index on the Unconfined Compressive Strength, Stiffness and Microstructure of Cemented Colombian Sands

**DOI:** 10.3390/ma17215193

**Published:** 2024-10-24

**Authors:** Luis Carlos Suárez López, Jesús Alberto Alcalá Vergara, Yamid E. Nuñez de la Rosa, Alvaro Arrieta, Jair de Jesús Arrieta Baldovino

**Affiliations:** 1 Civil Engineering Program, Universidad de Cartagena, Cartagena de Indias 130015, Colombia; lsuarezl@unicartagena.edu.co (L.C.S.L.); jalcalav@unicartagena.edu.co (J.A.A.V.); 2 Faculty of Engineering and Basic Sciences, Fundación Universitaria Los Libertadores, Bogota 110231, Colombia; 3 Department of Biology and Chemistry, Universidad de Sucre, Sincelejo 700001, Colombia; alvaro.arrieta@unisucre.edu.co

**Keywords:** sands, soil cementation, porosity/cement index, unconfined compressive, stiffness, microstructure

## Abstract

Artificial cementation of granular soils results in improved stabilization, increased stiffness, and greater mechanical strength. The porosity index and volumetric cement content $\left(\eta /{C_{iv}}^{a}\right)$ is presented as a key measure to study the evolution of different soil stabilization types. However, this index had not been previously studied or adjusted for sands in Colombia. Therefore, this study evaluates the applicability of the $\left(\eta /{C_{iv}}^{a}\right)$ index on unconfined compressive strength $(q_{u}$) and stiffness ($G_{o}$), complemented by microstructural analysis, in four sandy soils from Luruaco (Atlántico), Lorica (Córdoba), Medellín (Antioquia), and Bogotá D.C. The soils were stabilized with Type III Portland cement in dosages of 3%, 5%, 7%, and 9%, and subjected to UCS, ultrasound, and SEM-EDS tests after a curing period of 7 days. It was found that increasing cement content results in higher $q_{u}$ values for the samples, and higher molding density also leads to higher $q_{u}$ values. Additionally, the grain size distribution influenced the adjustment of parameter “*a*”. In the sands from Bogotá and Lorica, with high uniformity, the value of “*a*” was 1.00. In contrast, mineralogy and particle shape played a predominant role in the sands from Medellín and Luruaco, where the coefficient of uniformity is higher, suggesting a possible inverse relationship between particle angularity and the value of “*a*”.

## 1. Introduction

The combination of soils with minimal amounts of binding chemicals, such as Portland cement, is an artificial improvement method that aims to reproduce the stable internal structure of naturally cemented or weakly bound soils. The goal is to promote the reuse of local soils and minimize construction costs. Artificial cementation of granular soils increases stiffness, peak strength, and brittleness. Additionally, it mitigates excessive displacement or settlement of shallow foundations, protects the slopes of earth dams, and prevents the liquefaction of loose granular soils in subbases and base layers of roads and airfields [1,2,3]. The mechanical behavior of cemented soils is influenced by various parameters, such as cement content, cement type, density, confining stress, grain size, and stress–strain history [4]. In geotechnical engineering, soils can be improved for use in pavement, foundation support, and slope protection by incorporating cementing materials and aggregates such as lime, cement, pozzolans, fly ash, construction and demolition waste (CDW), and industrial waste [5].

The objectives of improving soils (as foundation and construction materials) include increasing strength, reducing distortion under stress, compressibility, susceptibility to liquefaction, natural variability of borrow materials, water pressure (redirecting seepage), controlling shrinkage, swelling, permeability, and preventing harmful physical or chemical changes due to environmental conditions [6]. Soil-cement mixing involves artificially structured materials with a stable structure due to artificial bonds, which are the hydrated minerals from the cement coating the soil compositions. The advantages of adopting soil–cement include the strength and longevity achieved with these materials and the possibility of using part of the local soil in the mix, thus avoiding the need to borrow applicable materials and dispose of local soil in stockpiles [7].

The addition of Portland cement to the soil (soil–cement) and densification through compaction in low-bearing-capacity soil are effective methods that are part of current geotechnical engineering projects [8,9,10]. Due to the difficulties of in situ sampling, the mechanical characteristics of cemented soils are often studied using artificial samples prepared in the laboratory and cured with different cementing agents [11]. Horpibulsuk et al. [12] explored the effects of mixing cement with silty clay, identifying three modification zones: active, inert, and deterioration. In the active zone (3–12% cement), strength increased significantly due to the formation of cementing products and reduced porosity. In the inert zone (15–30% cement), no significant improvements in strength or changes in porosity were observed. In the deterioration zone (30–45% cement), the lack of water prevented proper cement hydration, reducing strength. This study highlights the critical balance necessary in soil stabilization, emphasizing that excessive cement can paradoxically reduce soil strength due to inadequate hydration conditions.

The amount of added ordinary Portland cement affects the physical and mechanical properties of artificially cemented sand; as the cement content increases, the cohesive strength $\left(c^{’}\right)$ and the angle of shear resistance $\left({∆\phi }^{’}\right)$ gradually increase [13]. The main variables controlling the properties and characteristics of artificially compacted cemented sand mixtures are the proportion of cement in the mix, the degree of compaction, and the curing time [14].

Despite the widespread use of Portland cement in local soil improvement, there is no dosage methodology based on rational criteria as there is with concrete technology, where the water/cement ratio plays a fundamental role in evaluating strength or stiffness [15]. Due to the absence of a methodology involving an index to establish a soil–cement ratio, Consoli et al. [16] proposed a rational dosage methodology for soil–cement mixtures. The authors demonstrated the relationship between unconfined compressive strength $\left(q_{u}\right)$ and the index $\left(\eta /{C_{iv}}^{a}\right)$, which relates the porosity of the compacted mix $\left(\eta \right)$ to the volumetric cement content $\left(C_{iv}\right)$, adjusted by an exponent “a”. These parameters are structured in Equation (1), which is of the power type. Additionally, the evaluation of initial stiffness at small deformations $\left(G_{o}\right)$ through the index $\left(\eta /{C_{iv}}^{a}\right)$ is very convenient due to the explicit consideration of the two key ingredients that control the design of artificially cemented soil mixtures: soil density and cement content [17].

(1) $$q_{u}\;⋁G_{o}=A{\left[\frac{\eta }{{\left(C_{iv}\right)}^{a}}\right]}^{-B}$$

Parameter A is influenced by the strength of cementation bonds and the sand matrix, with the latter being less influential than the cementation bonds [3]. There is a relationship between −B and “a”; both parameters are related to the soil matrix, and the relationship between them is approximately a ≈ 1/B [1]. The exponent “a” is related to porosity and the volumetric cement content; when (a=1), porosity and the amount of cement have an equivalent influence on the strength of the soil–cement mixture. For a value of (a<1), it indicates that porosity has greater relevance in the mixture, and for (a>1), it specifies that the cementing bonds significantly influence the strength [18].

In previous studies, this methodology has been applied using Equation (1) for different sands, employing Portland cement as a cementing agent. Consoli et al. [16] studied a type of soil classified as clayey sand (SC) from the Porto Alegre region in southern Brazil, performing unconfined compression and triaxial strength tests, in which they obtained R2=0.97 adjustments and a value of 0.28 for parameter “a”. Ríos et al. [19] worked with silty sand and, for unconfined compression tests, obtained a value of a=0.21 and a correlation coefficient of R2=0.98 for a fixed moisture value and varying density. Consoli et al. [20] experimented with two types of soils: the first was a silty sand (SM) from the Porto region, in northern Portugal, and a uniform fine nonplastic sand (SP) from the Osorio region, near Porto Alegre, in southern Brazil. For both soils, in both compressive strength $\left(q_{u}\right)$ and stiffness $\left(G_{o}\right)$ of the SM and SP sands, values of a=1.0 and a=0.21, respectively, were obtained, with correlation coefficients of R2=0.96 and R2=0.99 for $q_{u}$ and R2=0.92 and R2=0.89 for $G_{o}$. Baldovino et al. [21] studied the same effect on elastic sandy silt (MH) collected in the city of Curitiba, Brazil, obtaining a value of =for compressive strength using Type IV and II cement as the cementing agent, and an R^2^ = 0.94. Finally, Scheuermann and Consoli [22] implemented the methodology on a clayey sand (SC) from the Botucatu sandstone, extracted from a borrow pit near Porto Alegre (Brazil), obtaining a value of “a” of 0.28 for both unconfined compressive strength and a correlation coefficient of R2=0.98.

The influence of grain size on the mechanical behavior of cemented sand was studied by Zhang et al. [23], who used cement-based coral sand composites reinforced with polyvinyl alcohol fibers (FRCSCC). Through compression and microstructure tests, they concluded that as the particle size of the coral sand increases, unconfined compressive strength decreases by 26%, where compression failure exhibits ductile behavior and the elastic modulus decreases by 11%. Additionally, SEM images show that the coral sand particle sizes affect the uniformity of fiber dispersion and the fiber failure mode. Particle shape, mineralogy, and texture generally have a mutual effect on strength, particle breakage, and compressibility [24].

The growing concern for environmental sustainability demands better ways to utilize the waste generated by society. Therefore, engineering projects must evaluate and consider the best available options [25]. The economic, social, and environmental impact influences the application of Portland cement as the main option for a cementing agent. Ferrazzo et al. [26] studied the impact of stabilizing foundry sand waste (WFS) with binders based on alkali-activated waste and Portland cement (PC), finding that the total cost of using alkali-activated waste was higher than that of PC, mainly due to the high direct costs of the alkaline activator. However, in terms of sustainability and social cost, alkali-activated waste presented better indices than PC. Both stabilization solutions can be applied by professionals considering the impacts associated with their use in relation to technical requirements.

The application and study of new binders that reduce the carbon footprint is a topic of interest in the scientific community. However, while research and implementations in construction around the world are underway, ensuring the optimal use of Portland cement as a binder in the artificial cementation of sandy soils plays an important role in reducing CO_2_ emissions. Therefore, this research proposes to evaluate the effect of grain size and the porosity/binder index on the mechanical behavior (compressive strength and initial stiffness at small strains) of four Colombian sands when artificially cemented using Type III Portland cement (PC III), through the use of a rational method for soil–cement mixture design. The aim is to generate equations that can be used by professionals when selecting one of the soils from the open-pit mines involved in the study. This is one of the few studies to apply the rational methodology proposed by Consoli et al. [16] for the artificial cementation process in Colombian sands.

## 2. Materials and Methods

### 2.1. Materials

**Figure 1:**
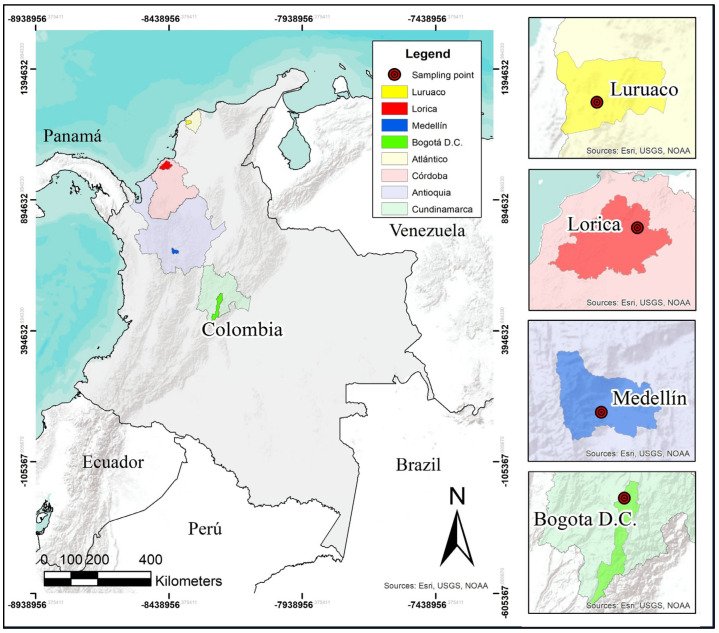
Map of origin of the sandy soil samples.

**Figure 2:**
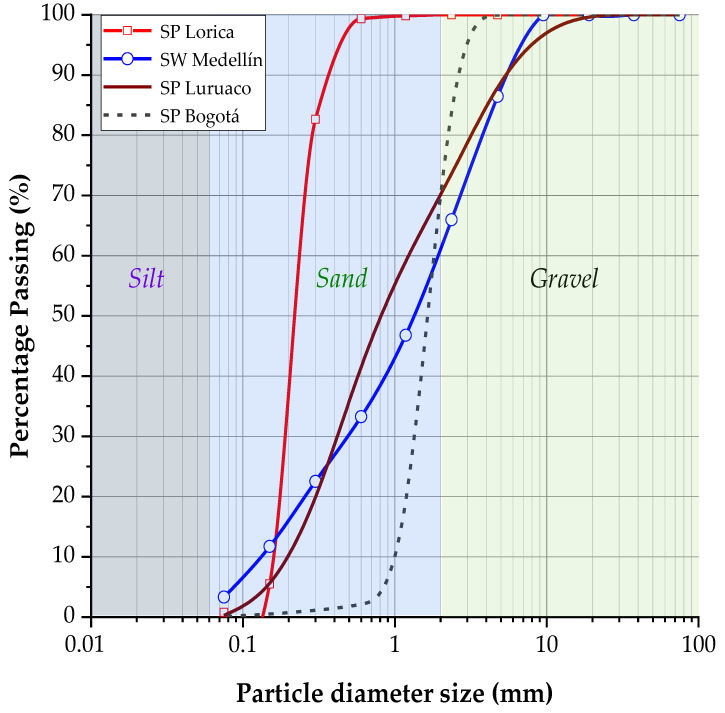
The granulometric curve of the soil sample, i.e., Lorica, Medellin, Luruaco, and Bogotá.

The sandy soil samples collected from various regions of Colombia (Figure 1), including sediments from the Sinú River in Lorica (Córdoba), samples extracted from an open-pit mine in southwest Medellín (Antioquia), material from an open-pit mine in Luruaco (Atlántico), and soils from Bogotá D.C., were the primary materials used in this investigation, along with Portland type III cement and water. After extraction, the samples were transported to Cartagena de Indias, where tests were conducted to determine their geotechnical properties, such as particle size distribution through sieving, specific gravity, and loose and vibrated densities. Additionally, an analysis of their elemental chemical composition was performed, and their microstructural properties were assessed using scanning electron microscopy (SEM) and energy-dispersive X-ray spectroscopy (EDS) by TESCAN, Brno, Czech Republic. The characterization results are presented in Table 1, while Figure 2 shows the particle size distribution curves of the soils. According to the Unified Soil Classification System, the uniformity coefficients of the clean sands from Lorica and Bogotá D.C. classify them as poorly graded sands. Similarly, the Luruaco soil sample falls into this classification, though it has a lower gradation coefficient. On the other hand, the Medellín soil sample is classified as SW.

The high initial strength Portland cement, classified as Type III according to ASTM C150 (2007) [27], is characterized by its rapid strength gain, which allowed for a seven-day curing period, ideal for the evaluation of soil–cement specimens [28]. The Specific gravity (*G_c_*) of cement is 3.11.

**Table 1:** Characteristics and properties of the soil sample (Lorica, Luruaco, Bogotá, and Medellin).

Property	Standard/Reference	Unit	Value			
			LOR	LUR	MED	BOG
Maximum γ_d_	[29]	kN/m^3^	1.56	1.78	2.02	1.70
Minimum γ_d_	[30]	kN/m^3^	1.37	1.52	1.63	1.55
Specific Gravity, Gs	[31]	-	2.73	2.73	2.83	2.69
Gravel (4.75–76.2 mm)	[32]	%	0	10.8	13.6	0
Coarse Sand (2.00–4.75 mm)	[32]	%	0	20.1	26.3	29.5
Medium Sand (0.425–2.0 mm)	[32]	%	5.1	42.3	35.5	69.4
Fine Sand (0.075–0.425 mm)	[32]	%	94.1	26.6	22.1	1.0
Silt (0.002–0.075 mm)	[32]	%	0.8	0.2	2.5	0.1
Mean Diameter (*d*_50_)	[32]	mm	0.24	0.85	1.38	1.75
Effective Diameter (*d*_10_)	[32]	mm	0.16	0.20	0.13	1.23
Uniformity Coefficient *C*_u_	[32]	-	1.6	5.9	14.6	1.4
Coefficient of Curvature *C*_c_	[32]	-	1.0	0.7	1.0	0.9
USCS Classification	[32]	-	SP	SP	SW	SP
Color	Munsell Chart	-	Dark greenish gray	Olive	Greenish gray	Pale yellow

The analysis of the elemental composition of four loose soil samples was performed using a scanning electron microscope (FE-MEB LYRA3 from TESCAN, Brno, Czech Republic) with an X-ray energy dispersive spectroscopy (EDS) microanalysis system, allowing us to obtain a detailed spectrum of the elements present and their relative percentage. Distribution maps of the elements detected in the analyzed areas were also generated. The scales used in the SEM were 2 mm, 200 microns, and 500 microns to improve the comparison of the characteristics of the samples. Quantitative analyses, with a precision of ± 2% and a detection limit of 100 ppm, allowed obtaining accurate results on the elemental composition of the soil samples.

**Figure 3:**
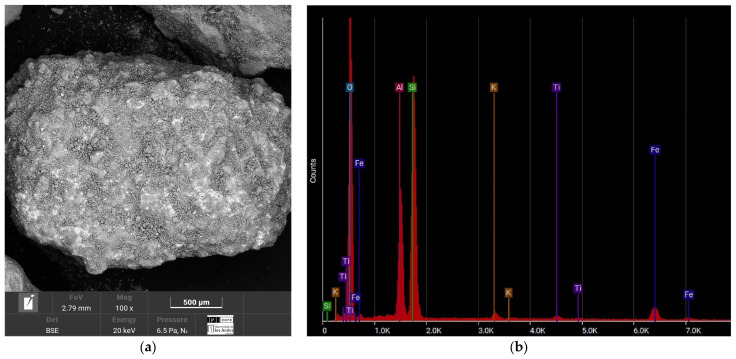
SEM-EDS test result of the Luruaco sands. (**a**) Microstructure. (**b**) Elemental composition.

Figure 3 shows the chemical composition of the Luruaco sand, classified as SP according to the USCS and coming from a mountainous area. This sand has a high aluminum content (13.18%) and silica (28.02%), classifying it as siliceous sand. The absence of calcium indicates that it is not carbonate sand, which could influence the interaction with Portland cement, limiting chemical reactivity but favoring a more stable matrix after cementation.

**Figure 4:**
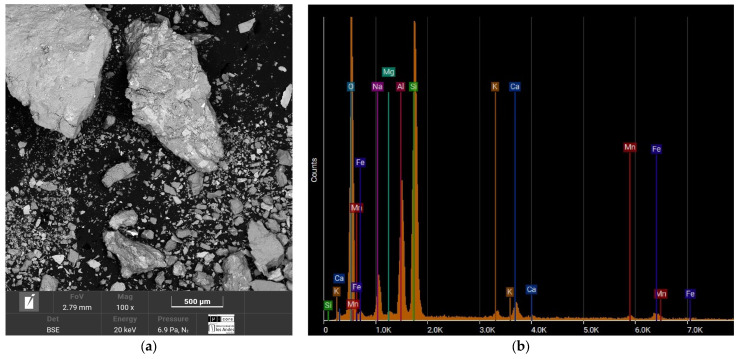
SEM-EDS test result of the Medellin sands. (**a**) Microstructure. (**b**) Elemental composition.

The Medellín sand in Figure 4 has a high silica content (28.49%) and a small proportion of calcium (2.41%) in its chemical composition. Its composition indicates siliceous sand with carbonate traces, which could influence its ability to react with cement during cementation, forming a stable structure with specific reactivity that can improve cohesion.

**Figure 5:**
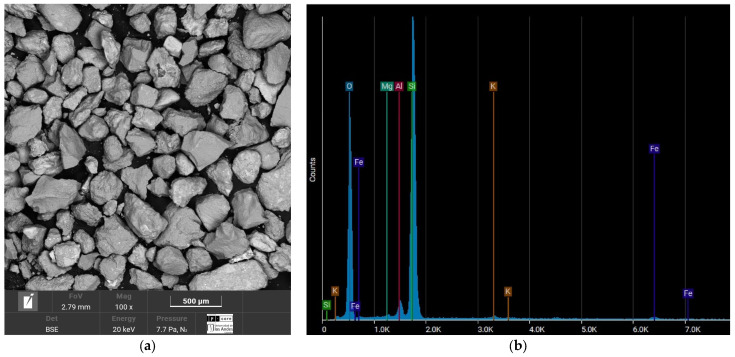
SEM-EDS test result of the Lorica sands. (**a**) Microstructure. (**b**) Elemental composition.

In contrast, the chemical composition of the sand from the Sinú River in Lorica (Figure 5) is characterized by its high silica content (43.76%). Classified as siliceous sand, it is suitable for cementing processes where chemical stability and low reactivity with cement are prioritized.

**Figure 6:**
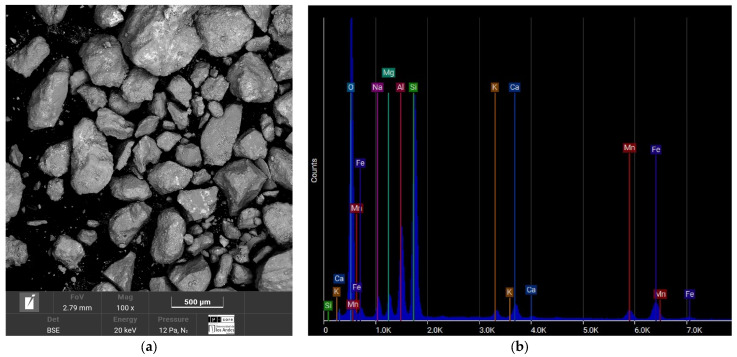
SEM-EDS test result of the Bogotá sands. (**a**) Microstructure. (**b**) Elemental composition.

Finally, the sand from Bogotá shows in its chemical composition in Figure 6 a moderate silica content (23.46%), together with calcium (2.33%) and iron (9.56%). This combination classifies it as a silica-carbonate sand, which makes it suitable for chemical interactions during cementation, with the potential to form a cohesive and resistant matrix.

### 2.2. Methods

The artificially cemented specimens were prepared using different cement contents and varying their densities, while maintaining a constant moisture content of 10%. The specimens were cured for seven days in a humid chamber to ensure proper hydration and hardening. Nondestructive ultrasonic testing was performed after a 24 h saturation period to assess their internal structure. Additionally, unconfined compressive strength tests were conducted under the same saturated conditions, along with microstructural analysis and chemical microanalysis to examine the composition and bonding characteristics of the cemented specimens.

#### 2.2.1. Specimen Molding and Preparation

Table 2 presents the detailed distribution of the samples, allowing for 144 specimens: 36 for each type of sand, 9 for each sand–cement content, and 3 for each combination of sand, cement content, and molding density point. A total of 144 specimens were fabricated, evenly distributed among the four sands studied. The selected cement percentages adhered to the guidelines of ACI 230 [33], which recommends a cement content for sands between 3% and 11%. In this case, four cementation percentages (3%, 5%, 7%, and 9%) were employed in combination with three intermediate levels of dry density, positioned between the loose dry density and the maximum density achieved through vibration, as established by the relevant standards [27,28].

The moisture content was maintained constant at 10% of the dry weight of the mixture, a value identified as optimal for sand cementation in previous research [24,26]. For the cementation process, Type III Portland cement was used, and a uniform curing time of seven days was established for all samples. The samples were removed from the mold on the sixth day of curing, and their masses and geometries were recorded. Subsequently, the samples were submerged in water to saturate them and minimize the effects of suction [34]. Finally, the samples were superficially dried, and their masses were measured again to obtain the density in a saturated state. These variable combinations were designed to thoroughly analyze the microstructural properties, unconfined compressive strength, and stiffness against small deformations. It is noteworthy that compaction energy was not applied, as the material classification allowed for precise density control through mass and volume.

Following the recommendations of ASTM D1632 [35], the preparation of the specimens was conducted in three successive layers, ensuring consistent densities and homogeneity in each layer through careful control of mass and volume. The upper surfaces of layers 1 and 2 were scarified to improve adherence between them, minimizing potential weakness planes. Once fabricated, the specimens were cured in a humid chamber for seven days to ensure adequate hydration. Given that the materials used were granular in nature, manual compaction was performed through vibration, achieving a constant volume of 247.34 cm^3^.

A cylindrical mold with a diameter of 5.4 cm and a height of 10.8 cm was used, adhering to the 1:2 ratio specified in ASTM D1632 [35], which outlines the standard dimensions and preparation procedures for cylindrical specimens in compressive strength tests. To ensure the quality of the specimens, strict acceptance criteria were applied, limiting the maximum variation in diameter to 0.5 mm and in height to 1 mm relative to the target dimensions. This ensured the consistency and dimensional accuracy of the samples throughout the study.

#### 2.2.2. USC and Stiffness (Non-Destructive) Program: Effects of Porosity/Cement Ratio

At the end of the 7-day curing period, the specimens were removed from the saturation vessels, which were used to minimize the metric suction. This measure was taken to mitigate the potential impact of metric suction on the unconfined compressive strength, recognizing its significant influence on the mechanical properties of the specimens [2,7,36,37].

The equipment used to estimate the small strain shear modulus (*G_o_*) was the Pundit Lab Proceq. The shear wave was obtained by vibrating transducers following ASTM C597 [38]. Subsequently, the small strain shear modulus of the material was calculated by multiplying the square of the shear wave velocity by the density of each material.

Next, the specimens were subjected to unconfined compressive strength tests, following Method B of ASTM D1633-17 [35]. Method B, due to the height-to-diameter ratio (2:1), provides a more accurate measure of unconfined compressive strength from a technical point of view. This test was performed in a 50 kN multitest hydraulic press with a sensitivity of 0.1 kN, and the breaking load was applied at a rate of 1.00 mm per minute. For the specimens’ chemical composition and microstructure tests, loose soil samples and pieces of specimens tested with a cement content of 5% were taken for each type of sand studied.

To evaluate the effects of the porosity/cement ratio on the unconfined compressive strength and stiffness of the four artificially cemented sands cured for seven days, the porosity (η) of the samples and the volumetric cement content $\left(C_{iv}\right)$ were calculated accord to Baldovino et al. [21] using the Equations (2) and (3).

(2) $$\eta =100-100\left\{\left[\frac{\gamma_{d}}{1+\frac{C}{100}}\right]\left[\frac{1}{\gamma_{s}}+\frac{\frac{C}{100}}{\gamma_{c}}\right]\right\}$$

where C is the amount of cement added expressed as a percentage, $\gamma_{d}$ is the dry unit weight of the sample, $\gamma_{s}$ is the unit weight of the soil solids, and $\gamma_{c}$ is the unit weight of the cement solids.

The volumetric cement content $\left(C_{iv}\right)$ is defined as the ratio between the volume of cement and the volume of a specimen sample $\left(V_{s}\right)$, as shown in Equation (3), where $G_{c}$ is the specific gravity of the cement.

(3) $$C_{iv}=\frac{100\left\{\left[\left(\frac{V_{s}\gamma_{d}}{1+C/100}\right)\left(\frac{C}{100}\right)\right]/G_{c}\right\}}{V_{s}}$$

From this, Equation (1) was used to generate the dosage curves, where the parameters “A”, “*a*”, and “−B” were calculated using the nonlinear least squares optimization method.

#### 2.2.3. Microstructure and Microanalysis of Artificially Cemented Soil

Compositional analyses using scanning electron microscopy (SEM) were meticulously performed on selected points within the cemented samples containing 5% cement. Surface inspections of the compacted blends were conducted at various scales, achieving magnifications of up to 500 times, providing a detailed perspective of the material’s morphology and structure. Subsequently, smaller magnifications were employed for a more in-depth exploration of the interactions between the sand grains and the cement matrix, reaching 200 and 20 μm, revealing microcracks and porosity at the grain interfaces. These tests were carried out using the advanced FE-SEM LYRA3 system from TESCAN, Brno, Czech Republic; ensuring precision and reliability in acquiring structural images and data.

Additionally, the SEM was equipped with an integrated microanalysis system utilizing energy-dispersive X-ray spectroscopy (EDS), allowing for detailed spectra of the chemical elements in the analyzed area and their relative percentages. Elemental distribution maps showed a predominant concentration of silicon in the sand grains and calcium in the cement matrix, confirming the expected composition for this type of material. Quantitative analyses had a precision of ±2%, with detection limits of 100 ppm, ensuring a detailed and accurate interpretation of the microstructure, both at the chemical and morphological levels.

## 3. Results and Discussions

### 3.1. Effects of Porosity/Cement Content Ratio on the Strength of Soil-Cement Mixtures

**Figure 7:**
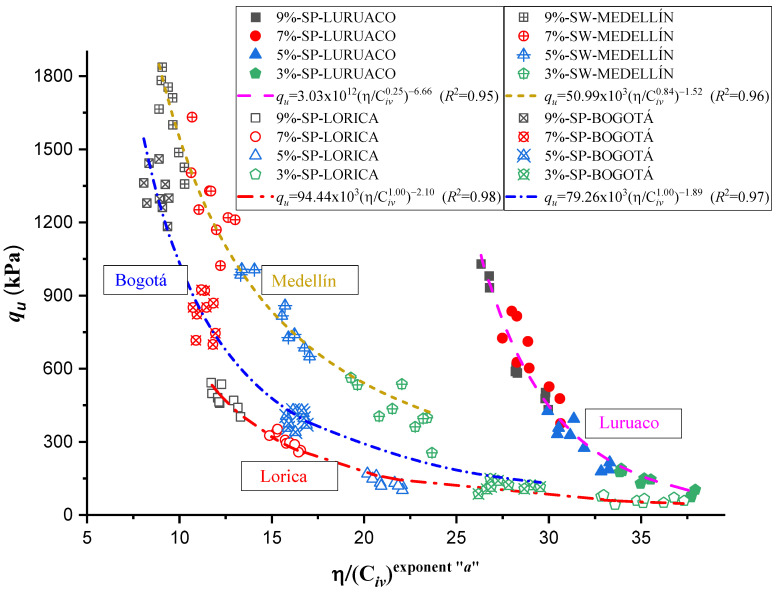
Effects of porosity/cement ratio on the unconfined compressive strength of 4 artificially cemented sands cured seven days.

Figure 7 shows the dosing curve that corresponds to the relationship between the *η*/*C_iv_* index and the unconfined compressive strength for each sand studied. Each trend line is based on the analysis of 36 specimens, with nine corresponding to each cement content. The relationship is expressed as the ratio between the specimen’s porosity and the volumetric index ($C_{iv}$), adjusted by an exponent “*a*”, independently calculated for each case, as shown in Table 3.

Parameter B, according to various authors [39,40,41,42] links the maximum strength to the soil state parameter, and its value varies according to the particular characteristics of the soil and the cement used [43]. In this sense, it has been established that the value of B strongly correlates with the materials’ properties and their interaction with the cement. Furthermore, the theoretical derivation by Diambra et al. [1] on the behavior of artificially cemented granular soils has highlighted an essential relationship between B and the parameter “*a*”, noting that a can be expressed as the reciprocal of. This implies that as B increases, “*a*” it decreases, reflecting a direct influence of B on the strength characteristics of the soil.

Applying this relationship to the cases in the Table 3, it is observed that B values vary according to soil type, indicating that each sand type has a different response in terms of strength due to its intrinsic characteristics. For example, for Luruaco sand, where *a* = 0.25 and the value of B = 6.66 is significantly higher than in other soils, suggesting a greater sensitivity to variations in the volumetric cement content and the *η*/$C_{iv}$ state parameter. In contrast, sands such as Medellín and Bogotá have lower values of B, suggesting less variability in strength concerning these parameters.

In this context, the theoretical relationship between B and “*a*” is primarily fulfilled since the variation of B in the different soils seems to influence the adjustment of the volumetric indices and the overall strength behavior.

The exponents for the sands from Luruaco and Medellín, i.e., 0.25 and 0.84, respectively, indicate that porosity had a more significant influence on compressive strength than the volumetric content of the binder [39]. On the other hand, for the sands from Bogotá and Lorica, both exponents, equal to 1.00, suggest that porosity and cement content had an equivalent influence on the strength of the soil–cement mixture. Regarding the analysis of the dosage curve, it is observed that the sand with the highest strength for the same cement content was the Medellín sand, a well-graded sand (SW) that also exhibited the highest molding density values, ranging from 19.5 to 17.5 kN/m^3^, which is due to its geotechnical characteristics. This density range and its grain size distribution allowed it to achieve the highest values of unconfined compressive strength (*q_u_*).

The results of the dosage curve show that the sand from Lorica exhibits the lowest strength values, along with the broadest range in the η/$C_{iv}$ index. This is associated with its high porosity and uniformity coefficient of 1.6, which indicates poor particle distribution. Additionally, Lorica contains the highest proportion of fine particles, with 94.1% falling within the acceptable sand range, according to Table 1.

The Equation (2) reflects the influence of the soil’s dry unit weight (γ*_d_*), the specific gravity of the solids, and the cement on porosity (*η*) [5]. A higher dry unit weight of the soil reduces porosity, which increases compressive strength by improving particle contact. Likewise, the values of *G_s_* and *G_c_* also affect this behavior; a higher *G_c_* (cement) tends to reduce porosity, resulting in a more compact and more substantial mixture. Therefore, the *η*/*C_iv_* index is crucial to understanding the relationship between porosity and compressive strength, as an increase in porosity generally reduces strength, while better cement distribution can mitigate this effect.

**Figure 8:**
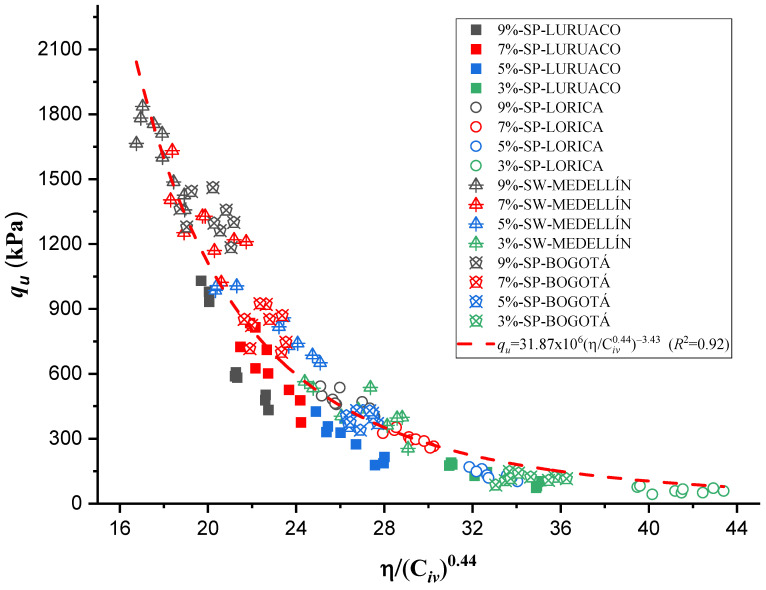
Effects of porosity/cement ratio on the unconfined compression of the 4 artificially cemented sands cured at 7 days in the same mathematical setting.

On the other hand, an adjustment was made for a single trend, considering all 144 tests, to obtain an ordinary prediction equation for the four sands studied. This adjustment yielded a value of *a* = 0.44 and a coefficient of determination (*R*^2^) of 0.92. The trend that higher cement percentages result in higher *q_u_* values remains consistent. The curve and the Equation are shown in Figure 8.

### 3.2. Effects of Porosity/Cement Content Ratio on the Stiffness of SoilCement Mixtures

**Figure 9:**
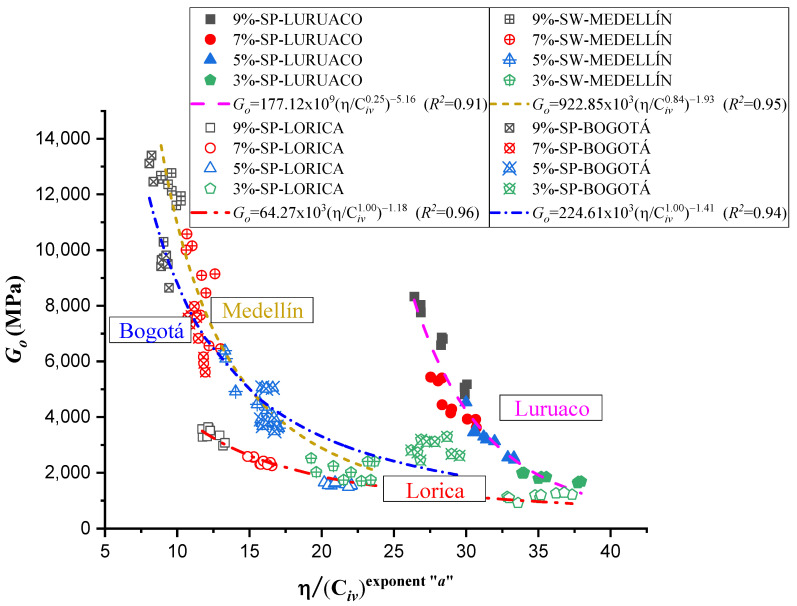
Effects of porosity/cement ratio on initial stiffness at small deformations in the four artificially cemented sands with 7 days of curing.

Figure 9 presents the stiffness results for the sands studied independently, highlighting a clear graphical trend compared to UCS values (Unconfined Compressive Strength). The adjusted “*a*” parameter, similar to the *q_u_* vs. *η*/$C_{iv}^{a}$, show consistent correlation. In the case of the *G_o_* vs. *η*/$C_{iv}^{a}$ ratio, correlation coefficients range from 0.96 to 0.91, as shown in Table 4.

**Figure 10:**
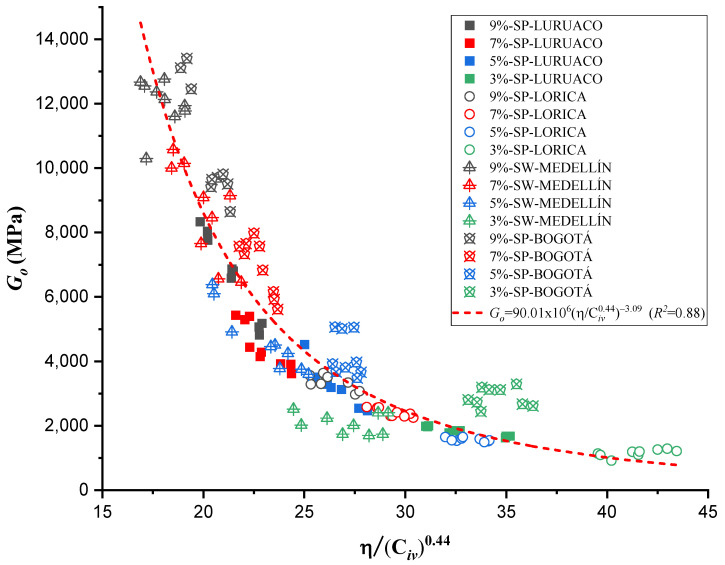
Effects of porosity/cement ratio on the stiffness of the 4 artificially cemented sands cured at 7 days in the same mathematical setting for a single adjustment value (0.44).

The equations in Figure 9 represent the power curve that relates the modulus *G_o_* to the values of *η*/ C*_iv_* for ages of 7 days and Figure 10 shows for a single adjustment value.

(4) $$G_{o}=A{\left[\frac{\eta }{{\left(C_{iv}\right)}^{a}}\right]}^{-B}$$

The results indicate that the relationship between cement content and shear modulus ($G_{o}$) varies significantly according to the type of sand, which is consistent with the existing literature on the mechanical behavior of stabilized soils. The Luruaco sand exhibits a volumetric adjustment coefficient of 0.25, suggesting a greater sensitivity to changes in cement content, possibly due to its classification as poorly graded sand, which limits densification. On the other hand, Lorica sand presents the highest coefficient of determination (*R*^2^ = 0.96), indicating that the fitted model accurately reflects the relationship between the volumetric index and $G_{o}$, highlighting the effectiveness of cement content in improving its stiffness. This finding is corroborated by the Medellín sand, which shows a fit coefficient of 0.84 and an *R*^2^ of 0.95, suggesting that the model is robust but with a lower dependence on cement content compared to poor sands. Likewise, the Bogotá sand, with a fit of 1.00 and an *R*^2^ of 0.94, demonstrates that a higher cement content can optimize particle interlock, thus increasing the strength of the material. These results underscore the importance of tuning parameters in modeling the stiffness of stabilized soils and the need to consider particle size characteristics and particle size distribution in the design of mixtures for geotechnical applications.

The cement improved several soil properties, increasing the unconfined compressive strength by increasing the volumetric cement content and reducing the porosity. Also, the stiffness showed similar increasing behavior, adjusting the particle size distribution more efficiently [44]. In addition, studies have highlighted that the initial stiffness can decrease under high confining stresses, changing from a dilatant and brittle behavior to a more compressible and ductile one [45,46,47].

### 3.3. Normalization of Unconfined Compressive Strength and Initial Stiffness at Small Deformations

Normalization, also known as the division of strength and stiffness, is used to find an appropriate equation to estimate $q_{u}$ and $G_{o}$ as a function of the normalized index $\eta /{\left(C_{iv}\right)}^{a}$ with a single potential trend for the four studied sands. According to Consoli et al. [48], in order to find a normalized equation, it is first necessary to determine all the normalization strengths and stiffnesses using a particular value of $\eta /{\left(C_{iv}\right)}^{a}=\nabla$. The particular value of ∇ for normalization can be chosen within the range reported in this research, between 5 and 45, for both unconfined compressive strength $\left(q_{u}\right)$ and initial stiffness at small strains $\left(G_{o}\right)$. For this study, a value of $\eta /{\left(C_{iv}\right)}^{a}=\nabla =28$ was taken for the equations governing $q_{u}$ and $G_{o}$ (shown in Table 3 and Table 4, and Figure 7 and Figure 9) to calculate the normalization strengths for $q_{u-n}$ and the normalization stiffnesses $G_{o-n}$ for each studied sand.

The normalization strengths for $q_{u-n}$ are 707.89 kPa (Luruaco), 86.03 kPa (Lorica), 324.3 kPa (Medellín) y 147.66 kPa (Bogotá). Meanwhile, the normalization stiffnesses for G_(o-n) are 6054.57 MPa (Luruaco), 1249.91 MPa (Lorica), 1507.89 MPa (Medellín) and 2058.55 MPa (Bogotá). After calculating the normalization strength and stiffness values, the unconfined compressive strength and initial stiffness at small strains for each specimen must be divided by the normalization strength and stiffness values for the corresponding type of sand (for both $q_{u}$ and $G_{o}$). Normalization is obtained by dividing Equation (1) by the specific and arbitrary values of compressive strengths and stiffnesses corresponding to a porosity value defined by $\eta /{\left(C_{iv}\right)}^{a}=\nabla$, which leads to:

(5) $$\frac{q_{u}}{q_{u-n}\left(\eta /{\left(C_{iv}\right)}^{a}=∆\right)}=\frac{A{\left(\eta /{\left(C_{iv}\right)}^{a}\right)}^{-B}}{A{\left(∆\right)}^{-B}}={∆}^{B}{\left(\eta /{\left(C_{iv}\right)}^{a}\right)}^{-B}\;$$

(6) $$\frac{G_{o}}{G_{o-n}\left(\eta /{\left(C_{iv}\right)}^{a}=∆\right)}=\frac{A{\left(\eta /{\left(C_{iv}\right)}^{a}\right)}^{-B}}{A{\left(∆\right)}^{-B}}={∆}^{B}{\left(\eta /{\left(C_{iv}\right)}^{a}\right)}^{-B}$$

**Figure 11:**
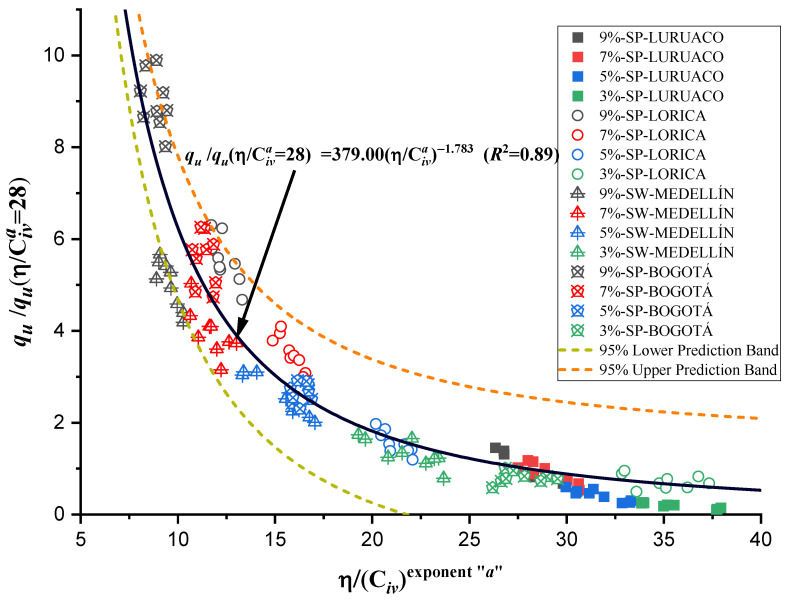
Normalization of unconfined compressive strength using the porosity/cement index.

This normalization was performed for 48 different sand–cement mixtures, with a total of 144 results for $q_{u}$ and $G_{o}$. The values of $q_{u}$ and $G_{o}$ used for the normalization can be found in Table 5. Figure 11 and the power type function of Equation (7) correspond to the best fit of the normalized strength values for the chosen value, with a coefficient of determination of 0.89, being a unique trend of all experimental and normalized points.

(7) $$\frac{q_{u}}{q_{u-n}\left(\eta /{\left(C_{iv}\right)}^{a}=28\right)}=379.00{\left(\eta /{\left(C_{iv}\right)}^{a}\right)}^{-1.783}\;\;\;\;\left(R^{2}=0.89\right)\;$$

(8) $$\frac{G_{o}}{G_{o-n}\left(\eta /{\left(C_{iv}\right)}^{a}=28\right)}=283.96{\left(\eta /{\left(C_{iv}\right)}^{a}\right)}^{-1.72}\;\;\;\;\left(R^{2}=0.81\right)$$

**Figure 12:**
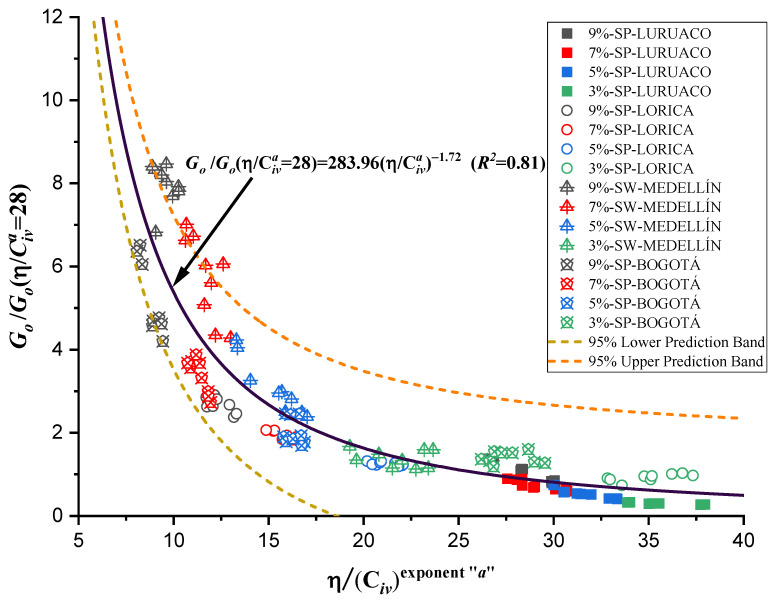
Normalization of stiffness data using the porosity/cement index.

Similarly, the initial stiffness data at small deformations $\left(G_{o}\right)$ were used to obtain Figure 12 and Equation (8), with a coefficient of determination equal to 0.81, which is lower than the adjustment for the unconfined compressive strength. However, despite presenting a lower adjustment, the normalization is valid to estimate the initial stiffness.

Equations (7) and (8) can be used for the different sands as cement content and porosity dosage ratios. If the equations are to be applied to obtain a strength or stiffness value required for a project, porosity, cement content, and one of the 4 sands studied must be selected. This way, the engineer can choose the optimum cement content that results in the most cost-effective option. Additionally, to increase the variability in the study of these sands, it is recommended that more studies be carried out using other types of cement and selecting various curing periods.

The behavior of unconfined compressive strength (Figure 11) and stiffness (Figure 12) present congruences when related to the index $\eta /{\left(C_{iv}\right)}^{a}$, in both cases as the index increases, the strength or stiffness decreases, which is understood as an inverse correlation. In addition, for each type of sand the different dosages used vary for the same volumetric content of cement.

**Figure 13:**
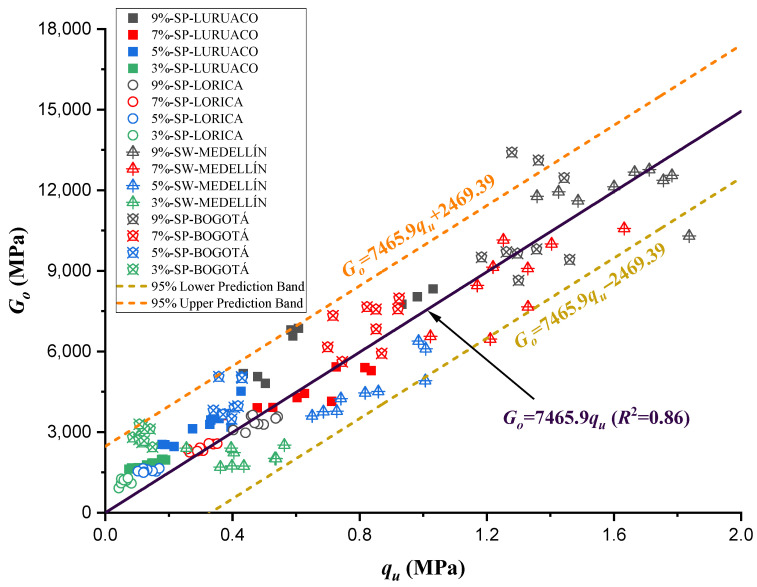
Direct relationship between the UCS and stiffness.

Figure 13 presents the direct relationship between strength and stiffness for all the specimen values studied. This relationship indicates that, as the resistance increases, the stiffness will also increase, being this relationship of linear character, as shown in Equation (9), with an *R*^2^ equal to 0.86. In this order of idea, the Medellín sample is the one that presents the highest stiffness and the highest compressive strength.

(9) $$G_{o}=7465.9q_{u}\;\;\;\;\left(R^{2}=0.86\right)\;$$

In Figure 13, the nonoriginal linear trend lines (prediction bands) support that there is stiffness in the sample even when there is no unconfined compressive strength, and the same happens for the opposite case, indicating the presence of additional factors that contribute to the presence of stiffness and absence of strength as the case may be. In addition, under the different sands studied, the case is presented where the stiffness is the same, but there are different resistance values. The opposite case is also present in the behavior that relates these two mechanical properties.

### 3.4. Microstructure and Microanalysis of Artificially Cemented Mixtures

**Figure 14:**
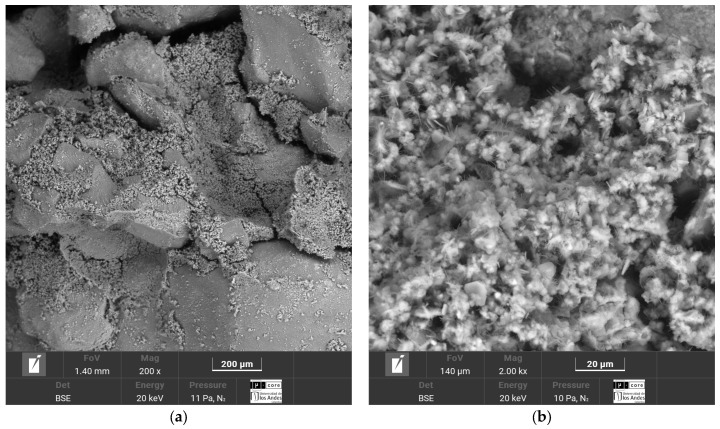
Microstructure analysis of Luruaco sand after 7 days of curing with a cement content of 5%. (**a**) Cementitious soil–cement matrix. (**b**) Formation of ettringite and C-S-H.

**Figure 15:**
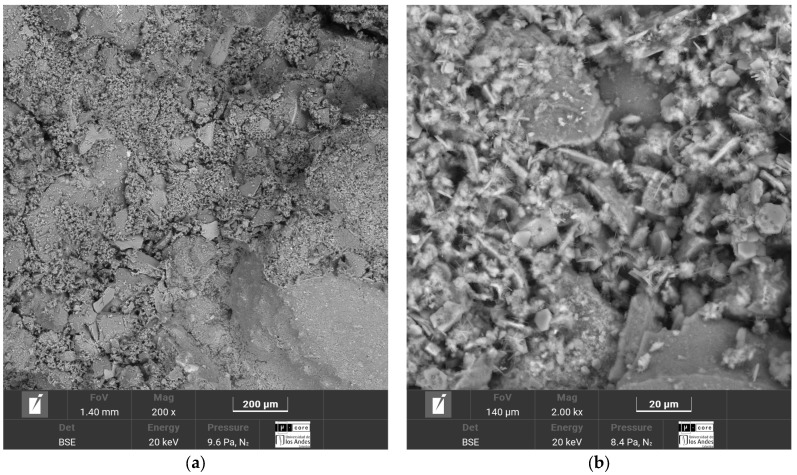
Microstructure analysis of Medellín sand after 7 days of curing with a cement content of 5%. (**a**) Cementitious soil–cement matrix. (**b**) Formation of ettringite and C-S-H.

**Figure 16:**
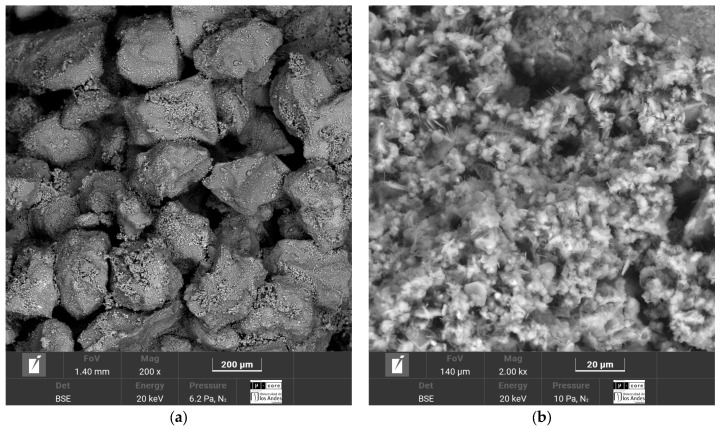
Microstructure analysis of Lorica sand after 7 days of curing with a cement content of 5%. (**a**) Cementitious soil–cement matrix. (**b**) Formation of ettringite and C-S-H.

**Figure 17:**
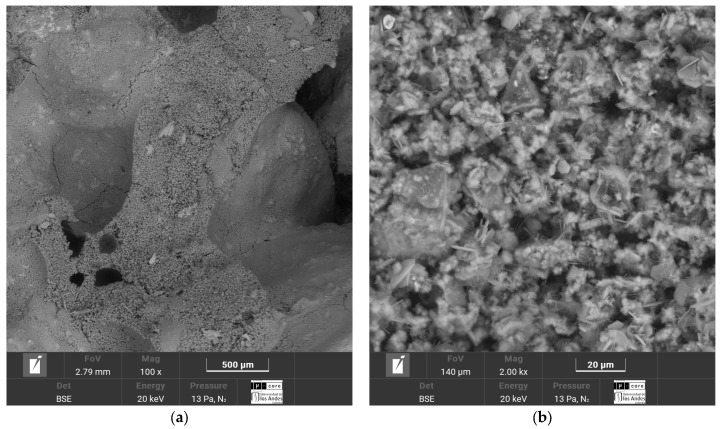
Microstructure analysis of Bogotá sand after 7 days of curing with a cement content of 5%. (**a**) Cementitious soil–cement matrix. (**b**) Formation of ettringite and C-S-H.

The SEM-EDS micrographs corresponding to the four soil–cement mixtures, Luruaco, Medellín, Lorica, and Bogotá, are presented in Figure 14, Figure 15, Figure 16 and Figure 17, respectively.

Figure 3b, Figure 4b, Figure 5b and Figure 6b reveals that the most abundant natural element in all the sands after oxygen is silicon (Si), so the sands can be considered quartzite. Elements such as oxygen (O), silicon (Si), potassium (K), aluminum (Al), and iron (Fe) are present in all four sands. One element to consider is calcium (Ca), due to its interference when the chemical reaction of the cement begins. This element was present in the sands of Medellín and Bogotá, although its percentage was the fourth lowest for Medellín and the second lowest for Bogotá.

The cementitious matrix between the soil and the cement allows for corroborating the strengths obtained for each sand (Figure 7 and Figure 9). In the case of Lorica (Figure 16a), there is the most outstanding amount of voids, where the particles are supported between them and the cement matrix; due to this high porosity, the Lorica soil showed lower strength and stiffness than the other soils. The sand of Luruaco and Bogotá (Figure 14a and Figure 17a) have fewer voids than the sand of Lorica, but the difference is in the cement matrix; Bogotá (Figure 17b) has a denser structure than Luruaco (Figure 14b). Therefore, the strength and stiffness of Bogotá were higher than those of Luruaco. Finally, in Figure 17a there are fewer voids, and the soil–cement matrix is denser, which suggests an improvement in structural integrity and mechanical properties.

From the microstructures of all the artificially cemented sands (Figure 14b, Figure 15b, Figure 16b and Figure 17b), it is evident that through the addition of cement, a chemical compound was generated that is evidenced as microcrystals in the form of needles called ettringite. These microcrystals present various dimensions and are dispersed throughout the soil–cement mixture. In addition, the presence of natural calcium in the sands (Bogotá and Medellin) and the calcium content in the cement causes the formation of a calcium silicate hydrate structure (C-S-H). These structures contribute to a more substantial confinement within the soil pore matrix. On the other hand, the reduction in the size and number of capillary channels within the matrix, understood as a reduction in capillary porosity, leads to a denser material formation and improves the structural and durability of the soil–cement mixture. The study by Baldovino et al. [48] presented similar characteristics in the cementitious matrix. They analyzed the behavior of silty soils by adding lime and glass powder. The result for the microstructure was the presence of hardened C-S-H and ettringite on the surface of the stabilized soil in the early stages of curing.

### 3.5. Influence of Grain Size and Mineralogy on the Porosity/Cement Ratio

The variation of the parameter “*a*” according to the type of soil is a consideration that has already been studied by different authors [16,19,20,21,22]; even in this research, it is proved that for the 4 treated soils the value of “*a*” presented 3 variations (0.25, 0.84 and 1.00). But according to Ríos et al. [49] the granulometric distribution explains part of the variation “*a*”, but the particles’ mineralogy and shape have the most decisive influence.

In Figure 2, the distribution curve of the Lorica and Bogotá sands presented a grain size distribution of small amplitude, which indicates, with the help of Table 1 uniform size among its particles with low $C_{u}$, while for the Luruaco and Medellin sands something different happens. Medellin has a wide range of particle sizes, and the medium particles are in adequate and balanced quantities. This is why this is the only well-graded sand (SW), while the Luruaco sand, despite having a better particle size distribution compared to the Bogotá and Lorica sands, $C_{u\;LUR}>C_{u\;BOG}\;y\;C_{u\;LOR}$, its curvature coefficient $\left(C_{c}\right)$ is the lowest of all, and it falls under the classification of poorly graded (SP), as do the other two. The relationship between the granulometric distribution and the value of parameter “*a*” has a particular effect that relates them in the cases where the $C_{u}$ was low (i.e., Lorica and Bogotá sands), the parameter “*a*” was the highest; for both cases, the adjustment presented a value of 1.00. Now, for the case of Luruaco and Medellin sands, the effect of the granulometry is not the central incident in the adjustment obtained according to parameter “*a*”, in both cases; this value was lower than the other two sands, but that of Luruaco (0.25) was lower than that obtained for Medellin (0.84), which is not governed by the relationship already mentioned for the cases where the value of the parameter was very high.

In this case, the elements that compose the two sands and the shape of their particles are the ones that indicate the value of the parameter. According to Figure 3a and Figure 4a, the particles of the Luruaco sand have a subrounded shape, while the Medellín sand is angular primarily. In addition, according to Figure 3b, Figure 4b, Figure 5b and Figure 6b, in both sands, the element with the highest presence after oxygen is silicon, which may indicate the presence of minerals such as quartz and feldspar. For this case study, the angularity is variable for the Medellin and Luruaco sands; the Medellin sand has mostly angular particles, and the parameter “*a*” was 0.85, while the Luruaco sand has fewer angular particles, and the adjusted parameter was 0.25. For this case study, it may be that the more angular the particles, the higher the parameter “*a*” than for the case where the particles are rounder. Establishing this relationship is arbitrary because only two sands were compared. Therefore, it is necessary to study other sands that present this difference in angularity to establish with greater experimental certainty how the behavior of “*a*” varies according to the shape of the particles.

## 4. Conclusions

The research carried out on stabilizing sandy soils with cement yields significant conclusions, although limited by the scope of the study. The main conclusions derived from the results obtained are presented below:

- The parameter “*a*” was the determinant in obtaining a good correlation coefficient (R^2^) in the four sands studied. The fits were satisfactory for the unconfined compressive strength ($q_{u}$) and the initial stiffness at small deformations ($G_{o}$). The equations obtained allow engineers to calculate the cement content and porosity required to achieve the desired mechanical properties in geotechnical engineering projects. Remarkably, *a* standard equation was obtained for all sands, using an “*a*” value of 0.44, showing a better fit than the normalization of individual equations (Figure 7, Figure 9, Figure 11, and Figure 12). This result facilitates the unification of criteria for similar soils in future projects, although its applicability should be verified in a broader range of sands.
- The sands’ grain size distribution impacted the parameter “*a*” adjustment. In Bogotá and Lorica, the “*a*” value was 1.00, which coincides with the behavior described in the literature for sands with a low coefficient of uniformity ($C_{u}$) (1.4 and 1.6, respectively). However, mineralogy and particle shape are predominant factors in the Medellín and Luruaco sands, where Cu is higher. The Medellín sand, with more angular particles (Figure 4), presented a higher “*a*” value, while the Luruaco sand, with more rounded particles (Figure 3), showed a lower “*a*” value. This suggests an inverse relationship between particle angularity and “*a*” value, but further studies on other types of sands are needed to corroborate this hypothesis.
- In all the sands, the cementitious matrix, with a cement content of 5%, maximum density, and 7 days of curing, presented minerals such as Ettringite and hydrated calcium silicate (C-S-H), responsible for improved mechanical strength. However, the Medellín sand showed a better distribution of the soil–cement matrix and a more significant reduction of voids (Figure 15), which explains its higher strength values. On the other hand, the Lorica sand presented a matrix with a greater presence of voids (Figure 16), which resulted in lower strength values. This behavior suggests that, in addition to the mineral composition, the distribution of voids is crucial for the performance of the soil–cement mixture. The differences observed in the microstructure of the sands point to the need to carefully evaluate the interaction between soil and cement for each specific case.
- In technical aspects, the equations generated from the research results will serve as a tool for professionals responsible for soil stabilization. With these equations, it will be possible to determine a value for the density and cement content of soils from the open-pit mines involved in the study, allowing the required strength to be achieved depending on the structure to be built on them. Additionally, applying the optimal cement content will reduce costs in soil–cement design and, furthermore, help decrease the carbon footprint by using the minimum amount necessary to meet the specifications of any civil engineering project.

## Figures and Tables

**Table 2:** Design combinations for the fabrication of cemented specimens.

Mix	Weight (%)		Curing Times (d)	Molding γ_d_ (kN/m^3^)	Number of Specimens
	Soil	Cement			
Luruaco soil–cement	100	3, 5, 7, 9	7	17.1, 16.5, 15.8	36
Medellin soil–cement	100	3, 5, 7, 9	7	19.5, 18.5, 17.5	36
Lorica soil–cement	100	3, 5, 7, 9	7	15.3, 14.8, 14.3	36
Bogotá soil–cement	100	3, 5, 7, 9	7	16.6, 16.2, 15.8	36

**Table 3:** Compressive strength equation.

Soil	Compressive Strength Equation	Adjustment of Volumetric Index “*a*”	Coefficient of Determination
Luruaco sand	$q_{u}=3.03\times 10^{12}{\left(\frac{\eta }{C_{iv}^{0.25}}\right)}^{-6.66}$	0.25	$R^{2}=0.95$
Lorica sand	$q_{u}=94.44\times 10^{3}{\left(\frac{\eta }{C_{iv}^{1.00}}\right)}^{-2.10}$	1.00	$R^{2}=0.98$
Medellin sand	$q_{u}=50.99\times 10^{3}{\left(\frac{\eta }{C_{iv}^{0.84}}\right)}^{-1.52}$	0.84	$R^{2}=0.96$
Bogotá sand	$q_{u}=79.26\times 10^{3}{\left(\frac{\eta }{C_{iv}^{1.00}}\right)}^{-1.89}$	1.00	$R^{2}=0.97$

**Table 4:** Compressive strength equation of compacted blends.

Soil	Stiffness Equation	Adjustment of Volumetric Index “*a*”	Coefficient of Determination
Luruaco sand	$G_{o}=177.12\times 10^{9}{\left(\frac{\eta }{C_{iv}^{0.25}}\right)}^{-5.16}$	0.25	$R^{2}=0.91$
Lorica sand	$G_{o}=64.27\times 10^{3}{\left(\frac{\eta }{C_{iv}^{1.00}}\right)}^{-1.18}$	1.00	$R^{2}=0.96$
Medellin sand	$G_{o}=922.85\times 10^{3}{\left(\frac{\eta }{C_{iv}^{0.84}}\right)}^{-1.93}$	0.84	$R^{2}=0.95$
Bogotá sand	$G_{o}=224.61\times 10^{3}{\left(\frac{\eta }{C_{iv}^{1.00}}\right)}^{-1.41}$	1.00	$R^{2}=0.94$

**Table 5:** Normalization data.

Soil Type	$\mathrm{Normalization}\;\mathrm{Index}\;\left(∆\right)$	For Normalization		$\mathrm{Coefficient}\;\mathrm{of}\;\mathrm{Determination}\;\left(R^{2}\right)$	
		$q_{u}\left(kPa\right)$	$G_{o}\left(MPa\right)$	$q_{u}\left(kPa\right)$	$G_{o}\left(MPa\right)$
Luruaco sand	$\eta /{\left(C_{iv}\right)}^{0.25}=28$	707.89	6054.57	0.95	0.91
Lorica sand	$\eta /{\left(C_{iv}\right)}^{1.00}=28$	86.03	1249.91	0.98	0.96
Medellin sand	$\eta /{\left(C_{iv}\right)}^{0.84}=28$	324.31	1507.89	0.96	0.95
Bogotá sand	$\eta /{\left(C_{iv}\right)}^{1.00}=28$	147.66	2058.55	0.97	0.94

## Data Availability

Data are contained within the article.
